# Eosinophilic Chronic Rhinosinusitis and Pathogenic Role of Protease

**DOI:** 10.3390/ijms242417372

**Published:** 2023-12-12

**Authors:** Jaehyeong Kim, Sooun Kwak, Juhyun Lee, Il-Ho Park, Seung Hoon Lee, Jae Min Shin, Tae Hoon Kim

**Affiliations:** 1 Department of Otorhinolaryngology-Head and Neck Surgery, College of Medicine, Korea University, Seoul 02841, Republic of Korea; kimwogud@gmail.com (J.K.); kwaksooun@gmail.com (S.K.); bm40461@naver.com (J.L.); parkil5@korea.ac.kr (I.-H.P.); shleeent@korea.ac.kr (S.H.L.); shinjm0601@hanmail.net (J.M.S.); 2 Mucosal Immunology Institute, College of Medicine, Korea University, Seoul 02841, Republic of Korea

**Keywords:** chronic rhinosinusitis, eosinophilic chronic rhinosinusitis, protease, endogenous protease inhibitor

## Abstract

Chronic rhinosinusitis (CRS) is an inflammation of the nasal and paranasal sinus mucosa, and eosinophilic CRS (eCRS) is a subtype characterized by significant eosinophil infiltration and immune response by T-helper-2 cells. The pathogenesis of eCRS is heterogeneous and involves various environmental and host factors. Proteases from external sources, such as mites, fungi, and bacteria, have been implicated in inducing type 2 inflammatory reactions. The balance between these proteases and endogenous protease inhibitors (EPIs) is considered important, and their imbalance can potentially lead to type 2 inflammatory reactions, such as eCRS. In this review, we discuss various mechanisms by which exogenous proteases influence eCRS and highlight the emerging role of endogenous protease inhibitors in eCRS pathogenesis.

## 1. Introduction

According to the European Position Paper on Rhinosinusitis and Nasal Polyps 2020 guidelines, eosinophilic chronic rhinosinusitis (eCRS) has emerged as a distinct subtype of chronic rhinosinusitis (CRS). This classification was based on observations from studies of patients with CRS with nasal polyps, particularly those who exhibited resistance to treatments and were prone to frequent relapses [1]. One defining characteristic of eCRS is the eosinophilic infiltration observed within the nasal tissue. Historically, eCRS was prevalent in the Western countries. However, recent trends indicate a rising incidence in Eastern countries, such as Japan, amplifying medical concerns in understanding and addressing this condition [2].

The etiology of eCRS is intricate and caused by a combination of external environmental triggers, such as allergens and pollutants, and internal host determinants, such as genetic predispositions and immune system responses. The nasal epithelium acts as the body’s primary defense barrier and responds to external threats by releasing cytokines, which are pivotal agents that stop the T-helper-2 (Th2) immune response. This cascade of reactions subsequently activates eosinophils, which is key in the inflammatory response to eCRS. However, the complete mechanistic pathway is yet to be elucidated and is currently being researched [3].

Proteases and proteolytic enzymes have been highlighted as potential agents that influence the pathogenesis of type 2 inflammatory diseases, including eCRS. These enzymes originate from external organisms, such as mites, fungi, and bacteria, and can induce immune reactions in predisposed individuals. The human body produces endogenous protease inhibitors as a defense mechanism to counteract these proteases. Current studies suggest that an imbalance between these proteases and their inhibitors may be important in the onset of type 2 inflammatory disorders, including eCRS [4].

Therefore, in this review, we explored the relationship between proteases and the pathogenesis of eCRS. Furthermore, we discuss the changes in the classification of chronic rhinosinusitis and review the diagnostic criteria and characteristics of eCRS. We provide an overview of proteases, including their definition and classification. We also address current research and information on the pathogenic role of proteases and protease inhibitors in eCRS.

## 2. Eosinophilic Chronic Rhinosinusitis (eCRS)

### 2.1. Classification of Chronic Rhinosinusitis

The inflammation of the nasal and paranasal sinus mucosae causes CRS. To diagnose CRS, symptoms such as nasal congestion, rhinorrhea, facial pain, and dysosmia should persist for at least 12 weeks. In 2012, CRS was classified into two subtypes based on the presence or absence of nasal polyps: CRS with nasal polyps (CRSwNP) and CRS without nasal polyps (CRSsNP) [5]. As studies on CRS have advanced, researchers have gained deeper insights into its underlying pathophysiology, clinical manifestations, and treatment responses. This heightened understanding has led to the emergence of new CRS subtype classification systems characterized by factors such as anatomical distribution, predominant endotypes, clinical illustrations, and phenotypic variations. In an updated classification system, CRS is categorized into primary and secondary CRS. When the disease is caused by airway inflammation, it is categorized as primary CRS. If the disease is secondary to other medical conditions, such as immunodeficiencies or autoimmune diseases, it is categorized as secondary CRS. Based on its anatomical distribution, it is further categorized into localized and diffuse types. In the localized type, the disease is confined to the sinonasal mucosa or occurs only on one side of the nose, whereas in the diffuse type, it affects both the upper and lower airways or occurs on both sides of the nose. This classification also distinguishes between type 2 and non-type 2, depending on their association with Th2 cells. Based on the clinical manifestations and phenotypic characteristics, CRS can be classified into more precise subtypes, such as eCRS, allergic fungal rhinosinusitis (AFRS), and central compartment atopic disease (CCAD), and each of them exhibits distinct clinical presentations and phenotypic traits [1,6].

### 2.2. Clinical Presentation, Diagnosis, and Treatment of eCRS

eCRS is a specific subtype of CRS characterized by significant eosinophil infiltration and Th2 immune response [3]. Patients with eCRS typically present with the primary symptoms of an impaired sense of smell and thick gel-like nasal discharge and multiple polyps on both sides of the nasal passage can be observed. These polyps are commonly found in the middle meatus and the olfactory clefts. Since opacification of olfactory cleft is associated with impaired olfactory function, mucosal swelling in middle turbinate and polyposis in middle meatus are assumed to be related to an impaired sense of smell in eCRS patients. Additionally, DNA trap derived from eosinophil extracellular trap cell death (ETosis), which comprises densely aggregated chromatins, is thought to be related to thick, mucinous discharge. [7,8,9,10,11,12]. When comparing the CT findings, the ethmoid sinus is more prominently affected than the maxillary sinus and exhibits a higher Lund–Mackay score than in non-eCRS [13,14,15]. There is a statistically significant association with conditions such as asthma, aspirin intolerance, and drug allergies in patients with eCRS, and it is thought that there is a common mechanism in the pathogenesis of these diseases [2,9,10,12,16,17].

The characteristics and diagnostic criteria for eCRS differ from other CRS subtypes. eCRS can be diagnosed based on the location and characteristics of the polyps, CT scan findings, blood test results, and concurrent associated conditions. According to the Japanese Epidemiological Survey of Refractory Eosinophilic Chronic Rhinosinusitis (JESREC) scoring system, if the disease is present on both sides, it scores 3 points; if there are nasal polyps, it scores 2 points; if shadows from the disease are more visible in the ethmoid sinus than in the maxillary sinus on a CT scan, it scores 2 points; if eosinophils in the peripheral blood are 2–5%, it scores 4 points; if they are 5–10%, it scores 8 points; and if they are more than 10%, it scores 10 points. eCRS is diagnosed when the total score is ≥11. Furthermore, the presence of a certain number of eosinophils when observing the paranasal sinus tissue or nasal polyps under a microscope at 400× magnification confirms eCRS diagnosis. However, the number of eosinophils per high-power field (HPF) that can be defined as mucosal eosinophilia varies between references. In Western countries, 5–10 eosinophils per HPF are considered mucosal eosinophilia. On the other hand, in Japan, 70–120 eosinophils per HPF are regarded as mucosal eosinophilia. This discrepancy is thought to arise from genetic and environmental differences between countries, as well as variations in the medications (corticosteroids and antibiotics) used in different research studies. Consequently, no global consensus exists regarding the specific eosinophil count required to diagnose eCRS [2,18,19].

The concept of eCRS was discovered and established through studies in Japan on patients who did not respond to endoscopic sinus surgery (ESS) or macrolide treatment [7,20]. As a result, this disease is characterized by being somewhat challenging to cure and does not respond well to treatment. ESS is the standard treatment for eCRS. However, there is a high recurrence of polyps after ESS, and when recurrence occurs, more radical treatment methods, such as reboot surgery or a modified Lothrop procedure, are required [21,22]. Oral corticosteroids can also be used to effectively treat patients with eCRS for reducing nasal polyps. However, there are no standardized guidelines for dosage and duration of use, and side effects can occur with its long-term usage and are therefore recommended for short-term usage. However, repeated and frequent use of short-term oral corticosteroid therapy has been reported to increase the risk of side effects [23]. According to the European Rhinologic Society Guidelines, biologic medication is recommended for patients with eCRS who frequently require oral corticosteroid therapy, experience side effects from corticosteroids, or show resistance to it [1]. Biologics drugs have been developed recently and have been reported to show good therapeutic effects on refractory eCRS. Anti-interleukin (IL) 4 receptor α antibodies are widely used, and other biologics are currently being developed [24,25,26]. However, some patients with eCRS do not respond to biologics, and there is a financial burden due to the high cost of the medication [27]. Additionally, when the medication is discontinued, the disease can recur, and thus, continuous administration is necessary [28,29]. Therefore, efforts are needed to find new treatment modalities to improve these drawbacks.

### 2.3. Pathogenesis of eCRS

Pathogenesis of eCRS involves various factors that contribute to the development and maintenance of inflammation. Although the exact mechanisms have not been completely elucidated, it is assumed that a combination of environmental (allergens, pollutants, and microbial agents) and host factors (genetic predispositions and immune responses) contribute to disease onset as an initial trigger [30]. Barrier dysfunction in the airway epithelium can increase the susceptibility to allergens or microbial agents. This dysfunction can be caused by genetic predisposition, environmental exposure, or both. A type 2 immune response can occur in the airway epithelium in response to these initial triggers [31].

As the first-line barrier, the nasal epithelium reacts to exogenous pathogens and environmental damage caused by physical or chemical irritants with receptors such as pattern recognition receptors (PRRs) or toll-like receptors (TLR). As a result of this reaction, IL-25, IL-33, and thymic stromal lymphopoietin (TSLP) are released and function as “alarmins” in response to environmental and microbial stimuli [32]. Moreover, TSLP, IL-25, and IL-33 play pivotal roles in initiating the Th2 immune response. Once produced by the epithelial cells in the nasal mucosa, these cytokines stimulate both type 2 innate lymphoid cells and Th2 cells, thereby facilitating the production of Th2 cytokines (IL-4, IL-5, and IL-13) [33,34,35].

IL-4 and IL-13 induce a class switch in B cells, thereby promoting IgE production. This, in turn, activates histamine secretion by mast cells and basophils [36,37]. Additionally, IL-4 and IL-13 impair the barrier function of nasal epithelium by affecting tight junction proteins. The barrier dysfunction induced by these cytokines results in increased airway epithelial permeability [38]. Stimulated by IL-13, goblet cell hyperplasia and increased mucin production occur in the nasal epithelium with upregulated mucin 5AC (MUC5AC) expression [39,40,41].

Macrophages are transformed into M2 macrophages by IL-4 and IL-13 [42]. M2 macrophages secrete factor XIIIa (FXIIIA), a transglutaminase that acts during the final stage of coagulation. FXIIIA produces a crosslinked fibrin mesh [43]. Tissue plasminogen activator (tPA) is produced in the nasal epithelium and plays a role in degrading fibrin. However, the secretion is reduced by Th2 cytokines. Therefore, fibrin deposition occurs due to FXIIIA secretion by macrophages and decreased tPA secretion by cytokines. Due to excessive fibrin deposition in the capillaries of the nasal mucosa, exudation of plasma and accumulation of plasma proteins in tissue occurs. This leads to tissue edema, elevated viscosity of nasal discharge, and the formation of nasal polyps [3,44,45].

**Figure 1:**
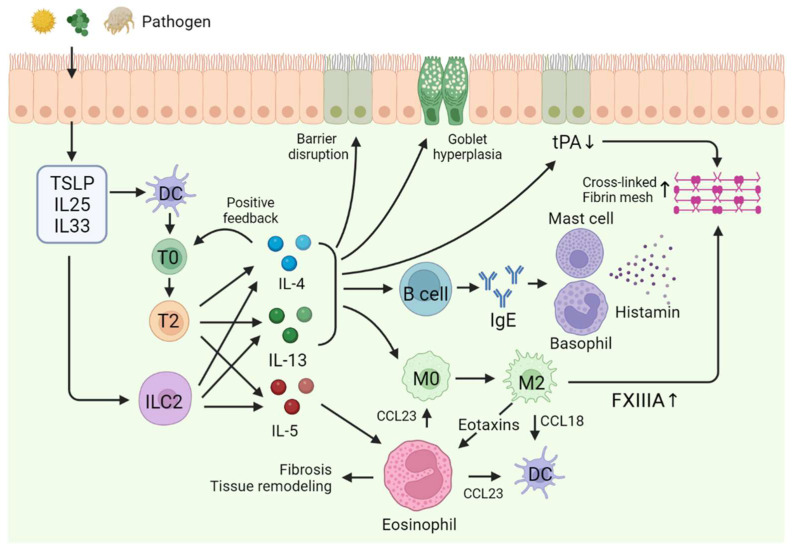
Pathogenesis of eosinophilic chronic rhinosinusitis. Abbreviations: TSLP, thymic stromal lymphopoietin; DC, dendritic cell; T0, naïve T cell; T2, T helper 2 cell; ILC2, type 2 innate lymphoid cell; M0, macrophage; M2, M2 macrophage; tPA, tissue plasminogen activator.

IL-5 is a key cytokine associated with eosinophils. IL-5 acts by activating the IL-5 receptor (IL-5R) in eosinophils. When IL-5 binds to IL-5R, intracellular signals related to eosinophil recruitment, activation, and survival are initiated. This interaction also spurs the formation of eosinophilic extracellular traps (EET) and the process of eosinophilic ETosis, leading to an increased viscosity in nasal discharge, as formerly mentioned. Additionally, this cascade results in the formation of Charcot–Leyden crystals (CLCs), which play roles in the attraction of neutrophils and promotion of inflammatory reactions, consequently amplifying disease severity [46,47,48]. Eosinophils contribute to the inflammatory response by interacting with other immune cells, such as T cells, B cells, and mast cells. Eosinophils contain granules comprised of proteins that can act as cytotoxins. By being stimulated with cytokines, eosinophils release these granule proteins and cause mucosal edema, nasal congestion, tissue remodeling, and recruitment of inflammatory cells. Furthermore, eosinophils enhance the expression of platelet tissue factor, which activates the coagulation cascade, thus contributing to excessive fibrin deposition in nasal mucosa and nasal polyp formation [46,47,48]. Studies suggest that when stimulated by IL-5, eosinophils produce CCL 23, which transforms macrophage into M2 macrophage. Furthermore, eotaxins (CCL11, CCL24, CCL26) and CCL 18 have been suggested to be secreted by M2 macrophages to recruit eosinophils and dendritic cells by creating a positive feedback loop for the activation of eosinophils [49]. Therefore, eCRS develops as a result of these interactions among different types of external stimuli, including inflammatory cells, cytokines, and chemokines. Its pathogenesis is heterogeneous, and many unknown mechanisms are yet to be elucidated (Figure 1).

## 3. Proteases

### 3.1. Definition and Functions of Protease

Proteases are enzymes that act as catalysts for breaking down proteins. They play various roles in various organisms. Proteases promote protein hydrolysis by breaking them down into amino acids and peptides of varying sizes. Proteases have specific functions and functions like “sharp scissors” in inducing proteolysis. Proteases regulate protein activity, control protein–protein interactions, and generate and play important roles in various signaling pathways. Alterations in the proteolytic functions of proteases are associated with the onset of various diseases. Therefore, proteases are often considered new drug targets or biological markers for diagnosis and prognosis prediction [40,50,51].

### 3.2. Classification of Proteases

Since P. A. Levene first described proteases in 1907, over 600 types of proteases have been identified in the human genome, and several more types of proteases have been discovered in living organisms. Proteases can be classified into seven types based on their catalytic mechanism and the nature of their active sites [52]. 

Serine proteases, such as trypsins, chymotrypsins, elastases, and protein C, contain serine residues in their active sites. They are important in various processes, such as digestion, blood clotting, and immune responses [53]. Cysteine proteases such as papain, cathepsins, and caspases possess nucleophilic thiol cysteine in their active sites and are primarily involved in senescence, apoptosis, and immune response [54,55]. Aspartic proteases such as pepsin, renin, and HIV-1 contain an aspartic acid residue in their active site. They play a role in the digestion and maturation of HIV; therefore, they are used as drug targets for treating HIV infection [56,57]. Metalloproteases such as matrix metalloproteinases (MMPs) and aminopeptidases require a metal ion, often zinc, for their catalytic activity. They are crucial for tissue remodeling and the degradation of extracellular matrix proteins [58,59]. Threonine proteases have a threonine residue in their active site, and proteasomal enzymes involved in the degradation of intracellular proteins belong to this class. Glutamic proteases such as scytalidoglutamic and aspergilloglutamic peptidases are less common and contain glutamic acid residues at their active sites. These proteases are primarily found in pathogenic fungi [60]. Asparagine peptide lyases are unique because they cleave asparagine residues from the N-termini of peptides. They do not utilize water for cleavage, distinguishing them from true hydrolytic enzymes [61]. Lastly, there are mixed and multiclass proteases. These proteases cannot be strictly classified into any of the above categories because they employ multiple residues or mechanisms. In addition to their mechanistic classification, proteases can be grouped based on their specificity, location (intracellular or extracellular), and physiological roles (Table 1).

### 3.3. Role of Proteases in Type 2 Inflammatory Diseases

Cysteine proteases, serine proteases, and other types of proteases can be found in mites, fungi, and bacteria and potentially act as allergens that trigger immune responses in susceptible individuals. For example, house dust mites (*Dermatophagoides pteronyssinus*, *Dermatophagoides farinae*) release at least four groups of major allergens that have serine and cysteine protease activities. Similarly, pollens from trees (e.g., Japanese cedar, birch) and weeds (short ragweed, Bermuda grass) contain at least one kind of serine or cysteine protease. These proteases have been implicated in the induction of type 2 inflammatory reactions upon exposure to the respiratory system or skin [62]. For instance, while *Staphylococcus aureus* is typically a commensal organism, it can become pathogenic in diseases such as asthma, chronic rhinosinusitis with nasal polyps, and atopic dermatitis. The pathogenesis mechanism involves not only allergic reactions induced by *S. aureus* enterotoxin-specific IgE but also the ability of *Staphylococcus aureus* protease-like proteins (Spls) to trigger a type 2 immune response. Particularly, *Staphylococcus aureus* serine protease-like protein D (SplD) can stimulate the secretion of IL-33 from airway epithelial cells, activating ILC2 and Th2 cells, thereby amplifying the type 2 immune response. Additionally, repeated exposure to SplD leads to the formation of SplD-specific IgE, contributing to the onset of type 2 inflammatory diseases [4]. To date, three mechanisms have been proposed to explain the initiation of type 2 inflammatory reactions by exogenous proteases.

Tight junction proteins, such as ZO-1, E-cadherin, and occludin, can be degraded by allergen-derived proteases, such as serine and cysteine proteases. After the degradation of tight junction proteins, the permeability of the nasal epithelium increases so that pathogens or allergens can easily pass through the nasal epithelium to the lamina propria and connective tissue. Kale et al. found that when the cockroach allergen Per a 10, which has serine protease activity, was administered to Calu-3 cells, there was degradation of tight junction proteins (ZO-1 and occludin) and an increase in the permeability of the nasal epithelium. In vivo studies also indicated an increase in IL-33, TSLP, and other inflammatory markers [63]. This degradation of tight junctions and enhanced inflammatory reaction due to exogenous proteases has been similarly observed in studies with allergens produced by house dust mites, tree or grass pollen, and airborne fungi in MDCK, Calu-3, and 16HBE14o- cells [62,63,64]. 

Proteases contribute to type 2 inflammatory diseases by activating the protease-activated receptors (PARs) in the airway epithelium. PARs are G protein-coupled receptors, and there are four types of PARs (PAR1, PAR2, PAR3, and PAR4). PAR2 is thought to play an important role in type 2 inflammatory disease [65]. On an ovalbumin challenge in a mouse model, eosinophil infiltration of mice lacking PAR-2 was downregulated. Conversely, eosinophil infiltration of mice with overexpression of PAR-2 was upregulated. In addition, deletion of PAR-2 decreased IgE levels 4-fold when compared with a wild-type animal. The extracellular portion of PAR2 contains N-terminal peptides that can be specifically cleaved by proteases. When cleaved by a protease, the cleaved part binds to extracellular loop 2 (ECL2) and initiates downstream intracellular signaling. On activation of PAR-2, cytokines, chemokines, and growth factors are produced, contributing to the induction of allergic inflammation with increased contraction of airway smooth muscle and promoted production of fibroblasts [65,66,67,68].

Fibrinogen cleavage products (FCPs) are generated by fibrinogen cleavage. FCPs stimulate toll-like receptor 4 (TLR4) and trigger the release of IL-13. In vivo experiments by Millien et al. provided evidence supporting the hypothesis that the generation of FCPs by protease instigates type 2 inflammatory disease [69]. Millien et al. administered protease from Aspergillus oryzae (PAO) intranasally to both wild-type mice and Tlr4^−/−^ mice. In the wild-type mice model, this led to airway hyper-responsiveness, upregulation of the mucin 5AC (MUC5AC) gene, and increased levels of cytokines (IL-4, IL-5, and IL-13). Conversely, these effects were markedly reduced or absent in the Tlr4^−/−^ mice model, indicating a significant difference in response based on TLR4 presence. Upon treatment with FCPs, C57BL/6 mice exhibited increased eosinophil infiltration and upregulation of the MUC5AC gene. However, this treatment did not lead to airway hyper-responsiveness or an increase in IL-4-secreting cells. Additionally, when these mice were treated with both PAO and hirudin, which has an anti-protease effect and inhibits fibrinogenolysis, the findings associated with allergic disease were mitigated in a dose-dependent manner. Furthermore, Cho et al. found that FCPs can stimulate mast cells to produce IL-13 in a TLR4-dependent manner [70].

**Figure 2:**
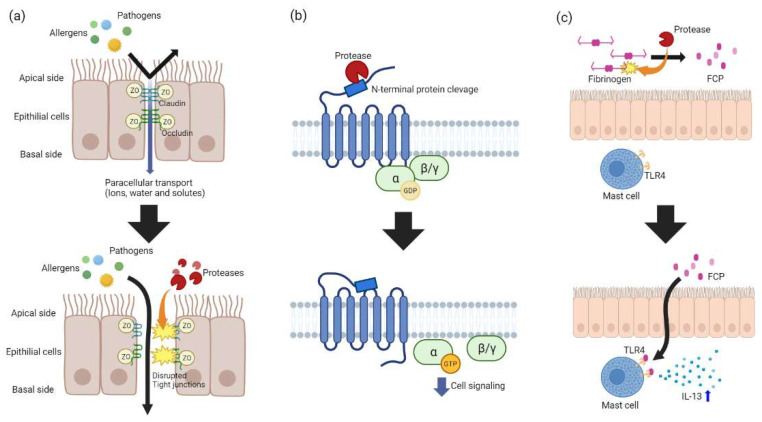
Role of proteases in the pathogenesis of type 2 inflammatory disease: (**a**) tight junction degradation and increased permeability of nasal epithelium by proteases; (**b**) PAR2 activation by cleaving N-terminal peptides by proteases; (**c**) TLR4 activation on mast cell by FCP and increased production of IL-13. Abbreviations: FCP, fibrinogen cleavage products; TLR4, toll-like receptor 4.

The onset of type 2 inflammatory diseases caused by exogenous proteases is inhibited or promoted by endogenous protease inhibitors. Studies on the relevance of type 2 inflammatory diseases and endogenous protease inhibitors regarding eCRS are being carried out. Notably, an imbalance between proteases and endogenous protease inhibitors (EPIs) is increasingly considered a major contributing factor to the onset of these diseases [4,71]. These studies suggest that targeting this imbalance could be a key strategy for treating and preventing type 2 inflammatory conditions (Figure 2).

### 3.4. Role of Protease Inhibitors in eCRS

The allergens found in house dust mites, fungi, and bacteria include cysteine and serine proteases. These proteases induce type 2 inflammation by disrupting tight junctions, activating protease-activated receptors, and stimulating TLR4 via FCPs. Because the onset of eCRS is associated with type 2 inflammation, these mechanisms related to exogenous proteases may play a role in eCRS [46]. However, recent studies have highlighted the role of endogenous protease inhibitors in the pathogenesis of eCRS, and the following paragraphs will discuss it in detail.

Cystatin SN, a member of the type 2 cystatin superfamily, acts as a protease inhibitor. It is suggested that cystatin SN inhibits tight junction disruption caused by proteases and histamine secretion by basophils in allergic rhinitis, which is caused by the Japanese cedar in an allergen-specific manner [72,73]. Fukuoka et al. [73] confirmed in an in vitro study that recombinant human cystatin SN (rhCystatin SN) inhibits the protease activity of Japanese cedar but does not inhibit the protease activity of ragweed. A similar allergen-specific phenomenon appeared to be present in ZO-1 degradation. In an allergen-sensitized mouse model, it was also observed that mice administered with rhCystatin SN showed a decreased frequency of sneezing in an allergen-specific manner. However, there was no significant difference in serum IgE levels between the Japanese cedar allergen-sensitized group and the ragweed allergen-sensitized group. However, in contrast to its regulatory effect on allergic diseases, cystatin SN is believed to contribute to the amplification of type 2 inflammation. It shows increased levels in nasal polyp tissue, and immunohistochemical staining has shown that its concentration increases with the severity of the disease and is often detected at higher levels than in non-eCRS [74]. There was a positive correlation between the increased mRNA expression of *TSLP*, *IL-33*, *CCL11* (eotaxin-1), and periostin and the increased expression of *cystatin SN* mRNA [75]. When stimulated with cystatin SN, the production of TSLP in the nasal mucosal epithelium increases, which could be due to a positive feedback loop between TSLP and cystatin SN. Similarly, the secretion of CCL11 and periostin by nasal mucosal fibroblasts increases when stimulated with cystatin SN. Additionally, cystatin SN secretion is enhanced by cytokines such as IL-4 and IL-13, while it appears to be inhibited by IL-17A. It also promoted the synthesis of eosinophil granule proteins through IL-5 [76]. Therefore, cystatin SN is presumed to be one of the factors that trigger eosinophil infiltration in eCRS. However, according to the study by Nocera et al. [74], it was discovered that in mouse models with ABCB1a mRNA knockdown, the stimulation of type 2 immune response by cystatin SN was reduced. ABCB1a mRNA is associated with the expression of p-glycoprotein, and p-glycoprotein is involved in the release of type 2 cytokines in a dose-dependent manner. These findings suggest a therapeutic approach to suppress the type 2 immune response induced by cystatin SN. 

**Figure 3:**
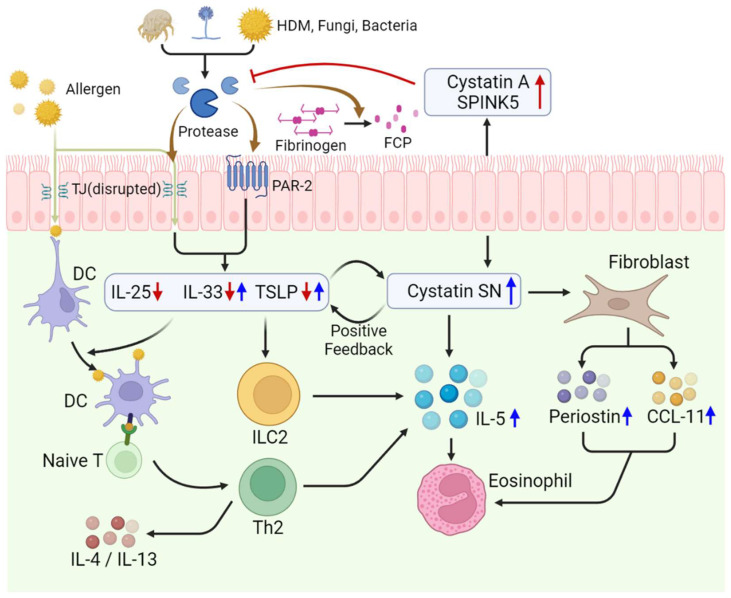
Pathogenic role of proteases and endogenous protease inhibitors in eosinophilic chronic rhinosinusitis. Red arrows signify the elevation of Cystatin A and SPINK5 and its results, indicating that their increase has a regulatory effect on other cytokines. Blue arrows represent the elevation of Cystatin SN and its results, denoting its role in promoting the increase of other cytokines and proteins. Abbreviations: HDM, house dust mite; TJ, tight junction; PAR-2, protease-activated receptor; DC, dendritic cell; TSLP, thymic stromal lymphopoietin; ILC2, type 2 innate lymphoid cell; Th2, T helper 2 cell; SPINK5, serine protease inhibitor Kazal-type 5.

Cystatin A and Serine protease inhibitor Kazal-type 5 (SPINK5) are endogenous protease inhibitors that regulate epithelial barrier function. According to Kouzaki et al. [71], the mRNA expression of *Cystatin A* and *SPINK5* is lower in eCRS than in non-eCRS. When recombinant Cystatin A and SPINK5 were administered to human nasal epithelial cells, the production of allergen-induced IL-25, IL-33, and TSLP decreased. Additionally, when Cystatin A and SPINK5 secretion was inhibited using small interfering RNA, there was an increased secretion of IL-25, IL-33, and TSLP. In a mouse model exposed to multiple airborne allergens, a decrease in the secretion and mRNA expression of *Cystatin A* and *SPINK5* was observed, along with inflammatory changes, such as goblet cell hyperplasia and epithelial disruption in the nasal epithelium. Conversely, when recombinant Cystatin A and SPINK5 were administered into the nasal cavity, allergen-induced pathogenesis was attenuated (Figure 3).

## 4. Conclusions

In this review, we discussed the roles of proteases and protease inhibitors in eCRS pathogenesis. Since the initial definition of eCRS was established, several studies have been conducted on its clinical characteristics, examination findings, pathogenesis, and treatment methods. However, to date, there is no global consensus on the histological diagnosis of eCRS. Its pathogenesis has not been fully elucidated, and its treatment method has not been fully established. In this review, we summarized the latest research and information on the pathogenic role of proteases and protease inhibitors. Proteases weaken the epithelial barrier, increasing allergen uptake by dendritic cells and activating the PAR-2 receptor or producing FCP, which in turn activates mast cells, thereby enhancing the secretion of cytokines and chemokines. This leads to the onset of type 2 inflammatory diseases. Among protease inhibitors, cystatin SN reduces allergic reactions in an allergen-specific manner but also increases the secretion of cytokines, triggering the pathogenesis of eCRS. Conversely, cystatin A and SPINK5 have been found to aid in the suppression of the disease. The effects and imbalances of these proteases and protease inhibitors on diseases need to be continuously researched in the future, and we hope that it provides new insights into the pathogenesis and treatment strategies of eCRS.

## Figures and Tables

**Table 1:** Classification of proteases based on their catalytic residues.

Class	Catalytic Residue	Examples	Functions
Serine Proteases	Serine	Trypsin Chymotrypsin Elastase Protein C	Digestion Blood clotting Immune response
Cysteine Proteases	Cysteine	Papain Cathepsins Caspases	Senescence Apoptosis Immune response
Aspartic Proteases	Aspartic acid	Pepsin Renin HIV-1 Protease	Digestion HIV maturation
Metalloproteases	Zinc	Matrix metalloproteinases (MMPs) Aminopeptidase	Tissue remodeling Wound healing Cell signaling
Threonine Proteases	Threonine	Proteasome enzymes	Intracellular protein degradation
Glutamic Acid Proteases	Glutamic acid	Scytalidoglutamic peptidase Aspergilloglutamic peptidase	Mainly found in pathogenic fungi
Asparagine Peptide Lyases	Asparagine	Viral capsid proteins Autotransporters of pathogenic bacteria	Mainly found in bacteria, virus

## Data Availability

Not applicable.
